# Alzheimer’s Disease Mutant Mice Exhibit Reduced Brain Tissue Stiffness Compared to Wild-type Mice in both Normoxia and following Intermittent Hypoxia Mimicking Sleep Apnea

**DOI:** 10.3389/fneur.2018.00001

**Published:** 2018-01-19

**Authors:** Maria José Menal, Ignasi Jorba, Marta Torres, Josep M. Montserrat, David Gozal, Anna Colell, Gerard Piñol-Ripoll, Daniel Navajas, Isaac Almendros, Ramon Farré

**Affiliations:** ^1^Unitat Biofísica I Bioenginyeria, Facultat de Medicina, Universitat de Barcelona, Barcelona, Spain; ^2^Institute for Bioengineering of Catalonia (IBEC), The Barcelona Institute of Science and Technology, Barcelona, Spain; ^3^Sleep Laboratory, Hospital Clinic Barcelona, Barcelona, Spain; ^4^CIBER de Enfermedades Respiratorias, Madrid, Spain; ^5^Department of Pediatrics, Section of Pediatric Sleep Medicine, Pritzker School of Medicine, Biological Sciences Division, The University of Chicago, Chicago, IL, United States; ^6^Department of Mort I Proliferació Cellular, Institut d’Investigacions Biomèdiques de Barcelona (IIBB-CSIC), IDIBAPS, CIBERNED, Madrid, Spain; ^7^Unitat Trastorns Cognitius, Clinical Neuroscience Research, IRBLleida-Hospital Universitari Santa Maria Lleida, Lleida, Spain; ^8^Institut Investigacions Biomediques August Pi Sunyer, Barcelona, Spain

**Keywords:** atomic force microscopy, brain mechanics, cortex stiffness, neurodegenerative disease, animal model

## Abstract

**Background:**

Evidence from patients and animal models suggests that obstructive sleep apnea (OSA) may increase the risk of Alzheimer’s disease (AD) and that AD is associated with reduced brain tissue stiffness.

**Aim:**

To investigate whether intermittent hypoxia (IH) alters brain cortex tissue stiffness in AD mutant mice exposed to IH mimicking OSA.

**Methods:**

Six-eight month old (B6C3-Tg(APPswe,PSEN1dE9)85Dbo/J) AD mutant mice and wild-type (WT) littermates were subjected to IH (21% O_2_ 40 s to 5% O_2_ 20 s; 6 h/day) or normoxia for 8 weeks. After euthanasia, the stiffness (E) of 200-μm brain cortex slices was measured by atomic force microscopy.

**Results:**

Two-way ANOVA indicated significant cortical softening and weight increase in AD mice compared to WT littermates, but no significant effects of IH on cortical stiffness and weight were detected. In addition, reduced myelin was apparent in AD (vs. WT), but no significant differences emerged in the cortex extracellular matrix components laminin and glycosaminoglycans when comparing baseline AD and WT mice.

**Conclusion:**

AD mutant mice exhibit reduced brain tissue stiffness following both normoxia and IH mimicking sleep apnea, and such differences are commensurate with increased edema and demyelination in AD.

## Introduction

Chronic intermittent hypoxia (IH), sleep fragmentation, and increased intrathoracic negative pressure swings are hallmark deleterious perturbations experienced by patients with obstructive sleep apnea (OSA). The oxidative stress and local and systemic inflammation elicited by the nocturnal cycles of hypoxia-reoxygenation experienced by patients with OSA (1, 2) are considered among the primary mechanisms triggering both the mid-term and the long-term adverse consequences of this sleep breathing disorder, namely increased risk of cardiovascular, metabolic, malignant, and neurocognitive diseases (3–7).

The prevalence of OSA, which is already considerable nowadays (8), is further anticipated to increase in the near future for the following reasons: (i) as a result of the current epidemic of obesity [one of the main risk factors for OSA (9)] in both developed and developing countries and (ii) aging of the population in light of the fact that OSA prevalence increases with age (8). Furthermore, population aging has potentially important implications for public health, since it has been suggested that OSA could boost the progression of Alzheimer’s disease (AD), one of the most relevant neurocognitive degenerative disorders associated with the aging process (10, 11). It has been proposed that the association between OSA and AD stems from the fact that both oxidative stress and inflammation are induced by OSA and enhance AD-related pathways (12–18). The existence of such an OSA-AD interdependence is supported by experimental data reporting that chronic IH boosts the expression of AD biomarkers and has negative neurocognitive impact in rodents (19), and by epidemiological studies showing a robust association between AD and OSA in humans (20, 21).

Intensive research efforts are currently ongoing aimed at implementing simple methods for early detection and long-term tracking of AD progression. In this context, one approach that has been recently proposed for this purpose is magnetic resonance elastography (MRE). This technique allows the noninvasive estimation of tissue stiffness in internal organs (22–24), and preliminary data indicates that AD is accompanied by reduced cerebral tissue stiffness in both animal models and patients (25–27). Accordingly, it has been proposed that brain tissue stiffness measured by MRE could be a mechanical biomarker for routine assessments of AD. Based on aforementioned considerations, we have recently found that IH exposures simulating OSA do not modify brain stiffness characteristics in healthy mice (28). However, whether an IH challenge affects the stiffness of brains with underlying ongoing AD-related processes—a most relevant question from the translational viewpoint—is still open. To further our understanding on the potential relationship between OSA and AD, the aim of the present study was to investigate whether chronic IH changes the mechanical properties of brain cortex in a well characterized mutant mouse model of AD. To this effect, we used atomic force microscopy (AFM) to directly and precisely measure the local Young’s modulus of brain cortex and investigated potential mechanisms that may account for some of the brain mechanical changes observed in these animals.

## Materials and Methods

### Animals

Alzheimer’s disease model mice (ADm; *N* = 16) of strain B6C3-Tg(APPswe,PSEN1dE9)85Dbo/J and their wild-type (WT; *N* = 27) littermates (all animals genotyped at the time of weaning) were used for the study. All mice (6–8 months old) were conventionally housed with water and food *ad libitum* and kept at 25°C under light-control (12L:12D). The experimental procedures were approved by the Ethics Committee of Animal Experimentation of the University of Barcelona following the local and European regulations in force.

### Application of Chronic IH

At the beginning of the experimental procedures, mice were randomly distributed into four groups: two groups for IH exposures (17 WT and 8 ADm mice) and two groups for normoxic room air (RA) (10 WT and 8 ADm mice). To apply IH, mice were placed in ~3 l boxes (26 cm long, 18 cm wide, 6 cm high) flushed with air with cyclic changes in oxygen content (40 s of RA at 21% O_2_ and 20 s of hypoxic air at 2% O_2_) with a rate of 60 events/h mimicking severe OSA. A zirconia solid-electrolyte cell oxygen sensor (MWL-F, 0.1–95% O_2_ ± 1%, Fujikura Ltd., Tokyo, Japan) was connected to the gas outlet of the box to continually measure the FiO_2_ in the chamber, showing that the air breathed by the mice ranged 21–5% O_2_. IH was applied for 6 h/day during the light period (10:00–16:00) for 8 weeks, with food and water freely available, as previously described (29). The mice subjected to RA were placed in similar boxes continuously flushed with normoxic air instead of hypoxic gas.

### Brain Tissue Sample Preparation

Mice were anesthetized with isoflurane and euthanized by cervical dislocation. Whole brains were extracted and weighted. For each brain the right and the left hemispheres were separated. From the right hemispheres, the cortices were isolated, weighted, and were then OCT-embedded. The left hemispheres were placed in ice-cold Krebs–Henseleit buffer modified and 200-µm thick coronal slices (between Bregma −1 mm and Bregma −2.5 mm) were obtained using a vibratome, and kept in ice-cold Krebs-Henseleit buffer until AFM measurements. The remaining tissue was fixed overnight with 4% PFA and paraffin embedded. All samples were code-labeled in a way that the operators involved in all the subsequent experimental processes were blinded to the sample group.

### Measurement of Brain Cortical Stiffness by AFM

The local stiffness of brain cortex was measured by AFM using a protocol described elsewhere (28). For each mouse, one brain slice was placed on the sample holder of a custom-built AFM mounted on an inverted optical microscope (TE2000; Nikon) within a 37°C-controlled environment. The slice was secured on the substrate inside a Petri dish by means of soft mesh of silicone thread (0.25 mm in diameter, 2 mm-spaced) in a ring. Then, dorsal cortical stiffness was measured in 9 points at the somatosensory area of the gray matter randomly selected and separated ~50–100 μm form each other. All measurements in each mouse were performed within 45 min after mouse euthanasia. A V-shaped Au-coated silicon nitride cantilever (0.01 N/m nominal stiffness) ended with a 12.5 µm radius spherical polystyrene bead (Novascan Technologies, Ames, IA, USA) was calibrated by the conventional thermal tune oscillation before cortical measurements. The piezoactuator-controlled vertical position of the cantilever (*z*) was measured with strain gage sensors (Physik Instrumente, Karlsruhe, Germany) and a quadrant photodiode (S4349, Hamamatsu, Japan) was employed to measure cantilever deflection (*d*). The relationship between the cantilever deflection and the photodiode signal was determined from a deflection–displacement *d–z* curve obtained in a bare region of the coverslip. The force (*F*) on the cantilever was determined as *F* = *k*·(*d* − *d*_0_), with *k* being the cantilever spring constant. The indentation magnitude δ was defined as δ = (*z* − *z*_0_) − (*d* − *d*_0_), with *d*_0_ and *z*_0_ being the offset of deflection and the displacement of the cantilever when the tip contacts the surface of the sample, respectively. The Hertz contact model for a sphere indenting a semi-infinite half space was employed: F=4E3(1−v2)R12δ32, with *R* = 12.5 μm being the radius of the bead, *v* = 0.5 the Poisson’s ratio and *E* the Young’s modulus. Using the previous equations for each force-indentation curve, *E, z*_0_ and *d*_0_ were determined by least-squares fitting considering maximum indentation values of ~1.5 μm. *E* for each measured point was computed as the average of the values obtained from 5 *F-z* curves (10 µm of ramp amplitude and 0.5 Hz of frequency; tip velocity of 10 µm/s).

### Visualization of the Brain Amyloid Plaque Deposition

Paraffin-embedded brain sections of 5 µm thickness were used to determine the presence of amyloid-β positive plaques. The tissue sections were deparaffinized, hydrated and incubated with 90% formic acid for 7 min at room temperature as antigen retrieval. To avoid unspecific binding, the slices then were blocked with 10% goat serum for 30 min at room temperature and incubated with the primary antibody solution overnight at 4°C. Mouse anti-β-amyloid clone 4G8 (800701, BioLegend) diluted 1:100 was used as primary antibody. Finally, the slices were incubated with goat anti-mouse-Alexa 488 (111-545-146, Jackson ImmunoResearch) as secondary antibody during 45 min at room temperature and mounted with Fluoromount-G (0100-01, SouthernBiotech). Nuclei were stained with NucBlue (R37605, ThermoFisher Scientific). Large epifluorescence images (10 images × 10 images) were obtained from each slice with the Nikon ECLIPSE Ti.

### Assessment of Glycosaminoglicans (GAGs) in Brain Cortex

Cortical frozen slices (20-µm thick) were stained with Alcian Blue to determine GAGs content. The cryosections were incubated overnight at room temperature with Alcian Blue solution [0.05% Alcian Blue 8GX (A3157, Sigma), 0.05 M sodium acetate and 0.1 M MgCl_2_ at pH = 5.7]. Subsequently, the slices were washed in deionized water, dehydrated by two changes of 95% ethanol and absolute ethanol (5 min each), and mounted with DPX (44581, Sigma). Color images of the cortex were obtained using optical microscopy (ZEISS Axiovert S100) and were analyzed with ImageJ software to compute the number of pixels of GAGs staining per μm^2^.

### Assessment of Laminin in Brain Cortex

Cortical frozen slices (20-µm thick) were used to assess laminin. Cryosections were defrost and permeabilized with 70% ice-cold methanol and 30% acetone for 5 min at room temperature and blocked with 10% fetal bovine serum for 30 min at room temperature to avoid unspecific binding and were incubated with the primary antibody solution overnight at 4°C. Rabbit anti-laminin (L9393, Sigma) diluted 1:200 was used as primary antibody. Finally, the slices were incubated with goat anti-rabbit-Alexa 488 (111-545-003, Jackson ImmunoResearch) as secondary antibody for 45 min at room temperature and mounted with Fluoromount-G (0100-01, SouthernBiotech). Nuclei were stained with NucBlue (R37605, ThermoFisher Scientific). Epifluorescence images from each cortical slice were obtained (Nikon ECLIPSE Ti) and analyzed with ImageJ software to compute the number of laminin-stained pixels per μm^2^.

### Assessment of Myelin Content in the Brain Cortex

Five-micron thick paraffin-embedded brain sections were stained with Luxol Fast Blue Stain (ab150675, Abcam) to assess their myelin content. The sections were deparaffinized, hydrated and incubated with Luxol Fast Blue Solution for 24 h at room temperature and subsequently rinsed in deionized water and differentiated in Lithium Carbonate followed by Alcohol Reagent. Finally, the slices were counterstained with Cresyl Echt Violet, dehydrated in absolute ethanol, and mounted with DPX. Color images along corpus callosum of each slice were obtained using optical microscopy (ZEISS Axiovert S100) and were analyzed with ImageJ software. To quantify the stained areas, the cyan color of each image was selected by using the Colour Deconvolution plugin of ImageJ software with the vector FastRed FastBlue DAB. The threshold intensity was manually set and kept constant in order to subtract the background of the staining and the Luxol Fast Blue positive pixels were determined to compute fold change in myelin content as described previously (30). All image analyses were performed by a single examiner who was blinded to sample group.

### Statistical Analysis

All values are expressed as mean ± SE. Two-way ANOVA were used to compare changes in cortical weight, brain weight, and *E* owing to treatment (IH vs. normoxia) and genotype (WT vs. ADm). *F*-values (*F*) and degrees of freedom (DF) from each variable are also provided. Student’s *t*-test was employed to compare histological staining between WT and ADm groups. Statistical significance was considered when *p* < 0.05.

## Results

**Figure 1:**
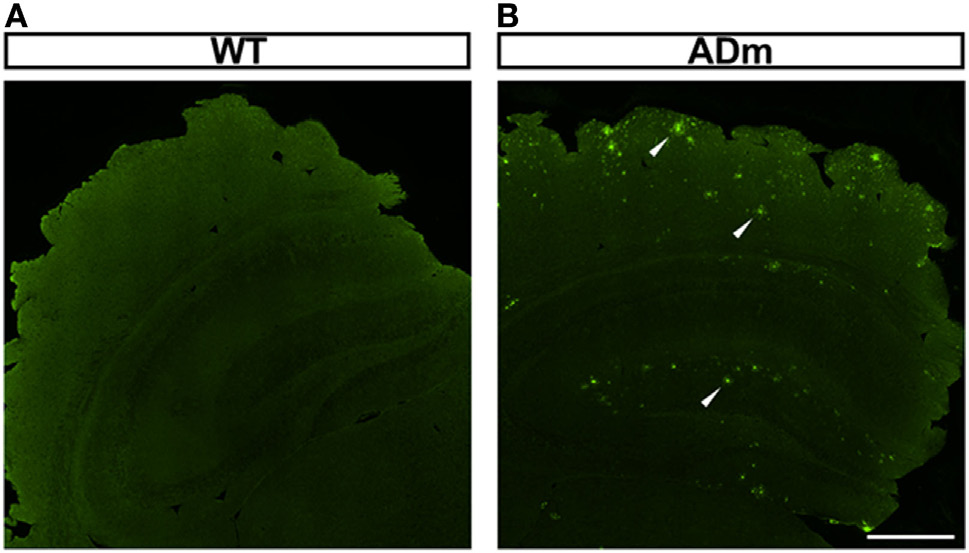
Illustrative example of amyloid-β positive plaque deposition. Coronal sections of paraffin-embedded brains from WT **(A)** and ADm **(B)** mice were stained using clone 4G8 immunosera. White arrowheads highlight the positive amyloid-β plaques deposited throughout the brain in ADm mice. Scale bar 500 µm. WT, wild type; ADm, Alzheimer’s disease transgenic mouse.

As expected from genotyping, amyloid-β positive plaques were observed in the cortex of all ADm mice and in none of the WT littermates, as illustrated by the examples shown in Figure 1.

**Figure 2:**
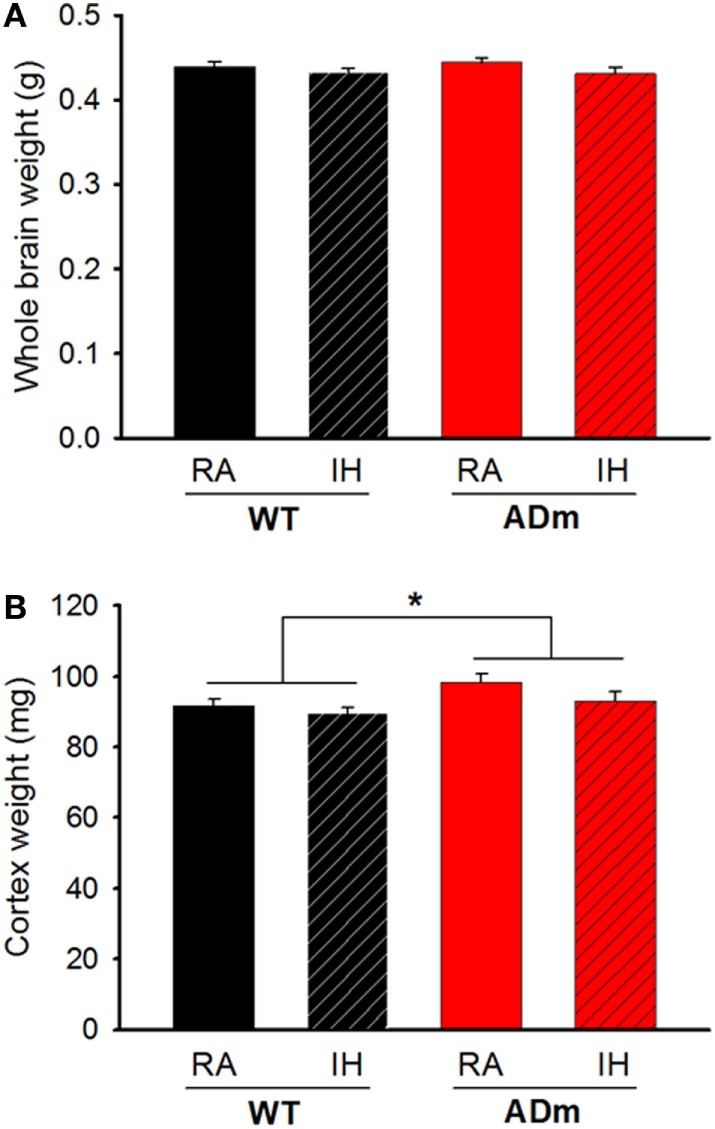
Cerebral and cortical weights. **(A)** Whole brain weights from the four experimental groups: WT mice subjected to RA (black bar) and IH (dashed black bar) and ADm mice subjected to RA (red bar) and IH (dashed red bar). Two-way ANOVA revealed no differences between experimental groups (IH vs. normoxia; DF = 1, *F* = 2.654, *p* = 0.111 and WT vs. ADm; DF = 1, *F* = 0.0992, *p* = 0.754). **(B)** Cortical weights from all the four experimental groups: WT mice subjected to RA (black bar) and IH (dashed black bar) and ADm mice subjected to RA (red bar) and IH (dashed red bar). Cortical weight was significantly higher in ADm mice compared to littermate controls (WT) (DF = 1, *F* = 6.042, *p* = 0.019). Two-way ANOVA analysis, *p < 0.05. Data are presented as mean ± SE. WT, wild type; RA, room air; IH, intermittent hypoxia; ADm, Alzheimer’s disease transgenic mouse.

No significant differences were observed in total brain weights for each factor (IH vs. normoxia; DF = 1, *F* = 2.654, *p* = 0.111 and WT vs. ADm; DF = 1, *F* = 0.0992, *p* = 0.754). However, cortex weight was significantly higher in ADm (98.3 ± 2.5 and 93.1 ± 2.5 mg in RA and IH, respectively) when compared with WT (91.6 ± 1.9 and 89.6 ± 1.6 mg in RA and IH, respectively) mice (DF = 1, *F* = 6.042, *p* = 0.019). No significant differences in brain and cortex weights emerged in IH-exposed mice when compared with the corresponding normoxic controls (DF = 1, *F* = 2.959, *p* = 0.094) (Figure 2). There was no statistically significant interaction between genotype (WT vs. ADm) and treatment (IH vs. normoxia) neither for brain (*p* = 0.618) nor for cortex weight (*p* = 0.449).

**Figure 3:**
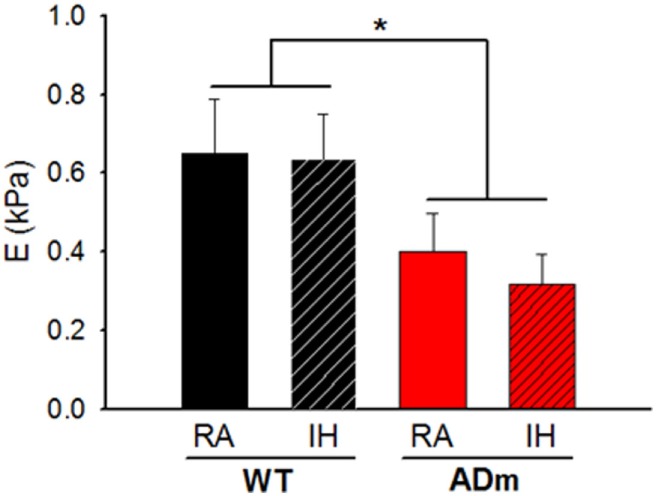
Stiffness of the cortex. The Young’s modulus (*E*) is a direct measurement of local tissue stiffness showing that cortical tissue was softer in ADm mice than in WT mice. *E* values from the four experimental groups, WT mice subjected to RA (black bar) and IH (dashed black bar) and ADm mice subjected to RA (red bar) and IH (dashed red bar). Cortical tissue stiffness was significantly lower in ADm mice vs. WT littermate controls (DF = 1, *F* = 6.198, *p* = 0.017). Two-way ANOVA, *p < 0.05. Data are presented as mean ± SE. WT, wild type; RA, room air; IH, intermittent hypoxia; ADm, Alzheimer’s disease transgenic mouse.

Figure 3 depicts the Young’s modulus (*E*) of the cortex which is a direct measurement of local tissue stiffness. This figure shows that genotype was a factor significantly modifying the local stiffness of brain cortex (ADm softer than WT) (DF = 1, *F* = 6.198, *p* = 0.017), which was not significantly altered by IH-exposures (DF = 1, *F* = 0.246, *p* = 0.623). In normoxia, *E* (mean ± SE; in Pa) was 402 ± 97 and 651 ± 138 in ADm and WT, respectively. After the IH exposures, *E* (in Pa) was 316 ± 78 and 637 ± 115 in ADm and WT, respectively. The ANOVA analysis revealed that there was no statistically significant interaction between genotype and treatment factor (*p* = 0.656).

**Figure 4:**
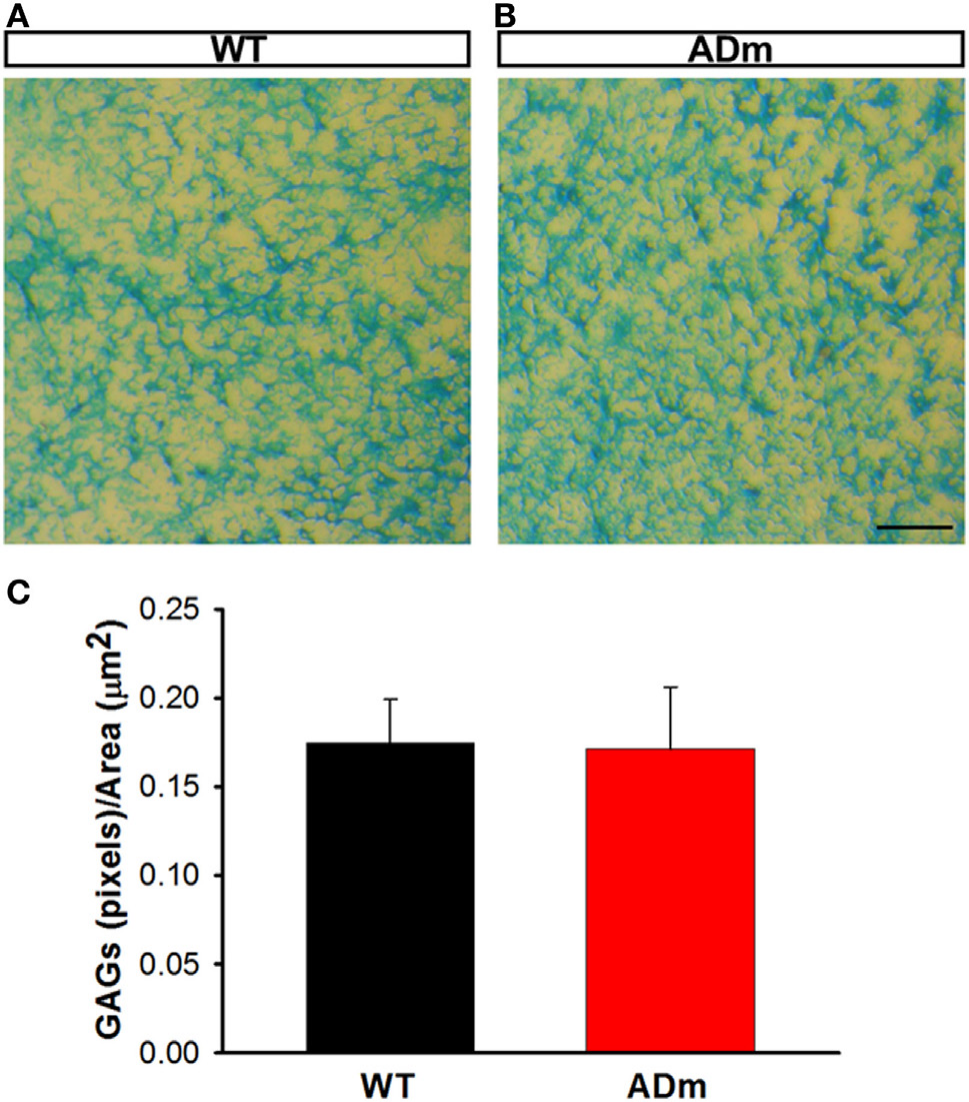
Cortical glycosaminoglicans (GAGs) staining. Cortical cryosections from WT mice **(A)** and ADm mice **(B)** subjected to RA exposures were stained with Alcian Blue. Scale bar 250 µm. **(C)** Quantification of the blue pixels (GAGs) per area (μm^2^) in WT tissue (black bar) and in ADm tissue (red bar) using Image J software. Using *t*-test analysis, no statistical differences between the two groups, WT vs. ADm, were found. Data are presented as mean ± SE. WT, wild type; ADm, Alzheimer’s disease transgenic mouse; RA, room air.

**Figure 5:**
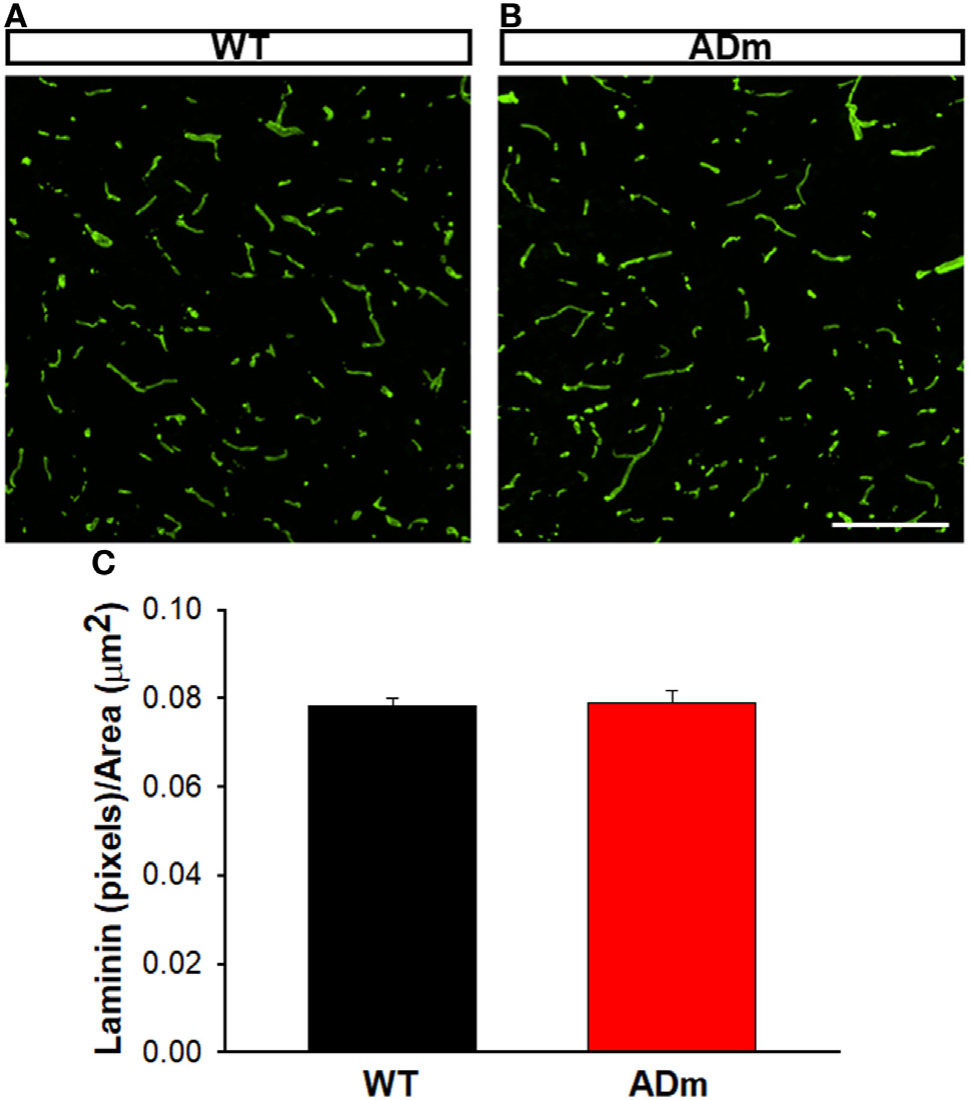
Cortical laminin staining. Cortical cryosections from WT mice **(A)** and ADm mice **(B)** subjected to RA exposures were stained with anti-laminin antibody. Scale bar 200 µm. **(C)** Quantification of the green pixels (laminin) per area (μm^2^) in WT tissue (black bar) and in ADm tissue (red bar) using Image J software. Using *t*-test analysis no statistical differences between the two groups, WT vs. ADm, were found. Data are presented as mean ± SE. WT, wild type; ADm, Alzheimer’s disease transgenic mouse; RA, room air.

**Figure 6:**
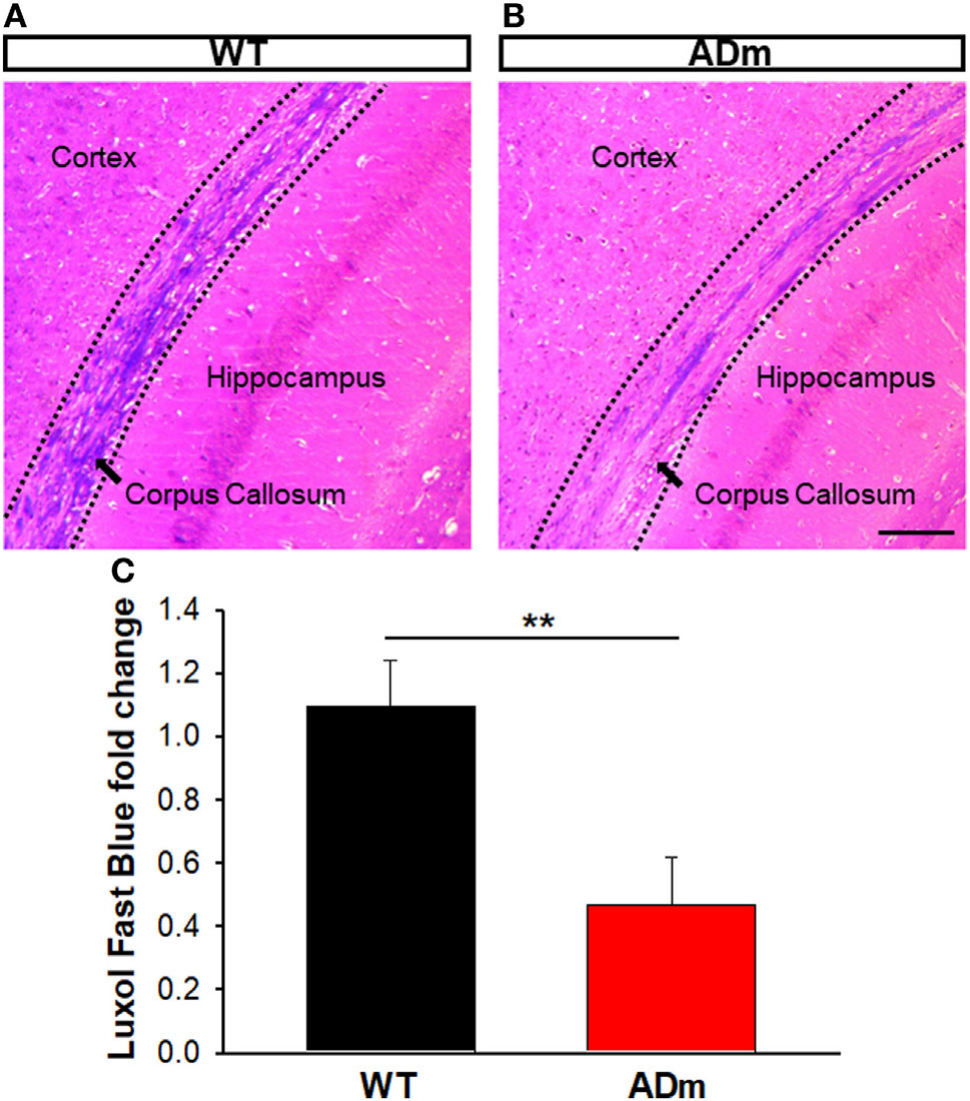
Myelin staining. Coronal sections of paraffin-embedded brains from WT mice **(A)** and ADm mice **(B)** exposed to RA were stained with Luxol Fast Blue. Scale bar 250 µm. **(C)** Quantification of the Luxol Fast Blue fold change (myelin) in WT tissue (black bar) and in ADm tissue (red bar) using Image J software. ADm mice showed a statistical significant decrease in the levels of brain myelin compared to the control mice, WT. *t*-Test, **p < 0.01. Data are presented as mean ± SE. WT, wild type; ADm, Alzheimer’s disease transgenic mouse; RA, room air.

No differences between ADm and WT emerged in the two most abundant extracellular cortical components, GAGs (Figure 4) and laminin (Figure 5). In contrast, ADm mice showed significantly less myelin content than WT littermates (Figure 6). Analysis of GAGs, laminin and myelin was carried out only in normoxic mice (five ADm and five WT) since IH did not modify cortical stiffness.

## Discussion

The emerging clinical interest of evaluating brain mechanical properties such as stiffness as a potential marker of AD, along with the cumulative evidence linking to mutually deleterious interactions between AD and OSA prompted the current study. Here, we focused on investigating whether application of chronic IH mimicking this sleep-disordered breathing adversely impacted on brain stiffness characteristics. Our findings showed that the brain cortex of ADm mice is considerably softer than their WT littermates, and that application of IH mimicking severe OSA did not modify cortical stiffness. The different stiffness found in ADm and WT mice is unlikely attributable to changes in extracellular matrix (ECM) components (laminin and GAGs) and is more likely accounted for the presence of edema, assessed by increases in cortex weight, as well as by demyelination. Thus, increases in overall water content and decreases in myelin emerge as potential mechanisms explaining the different mechanical properties of AD brains.

This study was performed by combining two conventional experimental models mimicking AD and OSA, respectively. First, a well-characterized mutant mouse strain of AD was employed. Among the several AD mouse models available (31, 32), we selected the B6C3-Tg(APPswe,PSEN1dE9)85Dbo/J mouse model—more commonly known as APP/PS1. This double transgenic mouse expresses a chimeric mouse/human amyloid precursor protein (Mo/HuAPP695swe) and a mutant human presenilin 1 (PS1-dE9), which are both directed to central nervous system neurons. Both of these mutations are associated with early-onset AD. In fact, this animal model is characterized by deposition of brain amyloid plaques (Figure 1) and with detectable and explicit neurocognitive alterations at the age of the mice used in this study (6–8 months) (33–35). Second, the model employed to mimic OSA was IH. The IH paradigm employed in this work (20% O_2_ 40 s to 6% O_2_ 20 s; 6 h/day; 8 weeks) simulates the hypoxia-reoxygenation events experienced by patients with considerably severe OSA readily reproducing the local and systemic effects observed in these patients. The model induces oxidative stress and inflammation, and augments metabolic, neurocognitive, cardiovascular and cancer consequences (1–3). In the brain, the IH exposures induce local events of hypoxia/reoxygenation (36, 37), promote activation and propagation of inflammatory and oxidative stress cascades (38–40), and lead to hypomyelination (41) and neuronal apoptosis (42–44), thereby resulting in neurocognitive and behavioral alterations (41, 45, 46). Importantly in the context of this study on AD, IH significantly increased the generation of β-amyloid peptides in 3xTgAD mutant mice harboring PS1M_146V_, APP_Swe_ and tau_P301L_ transgenes (19).

Here, we employed AFM to measure brain mechanical properties, since this technique allows us to directly measure the absolute value of the Young’s modulus (*E*) at well-defined sites in brain samples (47). Moreover, particular attention was paid to reduce the potential sources of experimental variability. To minimize the temperature dependence of measured stiffness (48), the brain tissues were kept in cold medium immediately after animal sacrifice, and also throughout the duration of the experimental processing of the samples, including manipulation and slicing. Moreover, cortex stiffness measurements were performed within a short time after euthanasia (49). The precision and resolution of AFM contrast with those provided by MRE. MRE is non-invasive and is based on image acquisition and processing using inversion model-based algorithms to derive a stiffness map of the organ (22–24). Not surprisingly, this complex process is associated with potential uncertainty (24, 50). In this context, the results obtained in the present study provide validation of MRE. Indeed, the precisely measured stiffness in this study shows reduced stiffness in ADm brains, thereby confirming the scarce previous data indirectly measured by non-invasive MRE in both AD animal models (25) and patients (26, 27). Of note, it is remarkable that the cortex stiffness reductions in ADm mice measured herein by AFM (21 and 38% in IH and RA, respectively) are quite consistent with the 22.5% reductions in brain stiffness previously measured by MRE in the same AD mutant mouse strain (25). Therefore, although it was not an aim of this study, the present results are of interest for clinical translation, since they provide strong support to the capability of MRE for detecting tissue changes in altered brains (51, 52).

The results in the present animal model work indicate that exposures to severe IH, eliciting oxidative stress and inflammation, do not modify the underlying alterations of cortical brain stiffness induced by AD. Given that the few previous studies available on brain mechanics in AD were observational (25–27), in the present work, we investigated potential mechanisms explaining brain softening in AD. In most body tissues, stiffness is mainly determined by its ECM, with elastin and collagen being the key components providing mechanical support. However, these two structural molecular species are almost absent in the brain, with laminin and GAGs accounting for the main ECM components (53). The fact that the abundance of both laminin and GAGs was not different in ADm and WT mice (Figures 4 and 5) suggests that the differences observed in cortical stiffness were not caused by AD-induced alterations in the tissue ECM. However, although not far from providing a definitive proof, the results reported in Figures 2 and 6 give clues to potentially explain brain tissue softening in AD. Indeed, the significant increase in cortical weight in ADm mice is suggestive of edema, which in fact has been reported in the AD brain (54, 55). In this context, increases in the proportion of water in the tissue are expected to reduce tissue stiffness (56). Moreover, the demyelination observed here in the ADm brains, which is characteristic in this disease (57, 58), is expected to further reduce tissue stiffness, particularly in light of the inverse relationship between brain stiffness and myelin content (59, 60).

## Conclusion

Chronic IH, a hallmark feature of OSA that characteristically induces oxidative stress and inflammation, does not modify the stiffness of brain cortex in normal mice, and similarly does not alter the mechanical properties of the cortex in a murine model of early-onset AD. Moreover, precise measurements of brain stiffness by AFM are supportive of previous evidence suggesting that MRE is a potentially valid and useful non-invasive mechanical marker of AD. The softening observed in AD mouse brain is likely attributable to edema and demyelination, although more research is required to better understand how AD and OSA modify brain mechanics.

## Ethics Statement

The experimental procedures were approved by the Ethics Committee of Animal Experimentation of the University of Barcelona following the local and European regulations in force.

## Author Contributions

MM, IJ, and MT: carried out most experiments. RF and IA: conceived the work and drafted the manuscript. DG, DN, AC, JM, and GP: analyzed the data. All authors discussed the work and finally contributed to the manuscript.

## Conflict of Interest Statement

The authors declare that the research was conducted in the absence of any commercial or financial relationships that could be construed as a potential conflict of interest.
